# Positron Emission Tomography/Computed Tomography in Polymyalgia Rheumatica: When and for What—A Critical Review

**DOI:** 10.3390/diagnostics14141539

**Published:** 2024-07-17

**Authors:** Elena Heras-Recuero, Teresa Blázquez-Sánchez, Laura Cristina Landaeta-Kancev, Marta Martínez de Bourio-Allona, Arantxa Torres-Roselló, Fernando Rengifo-García, Claritza Caraballo-Salazar, Raquel Largo, Santos Castañeda, Miguel Ángel González-Gay

**Affiliations:** 1 Division of Rheumatology, IIS-Fundación Jiménez Díaz, 28040 Madrid, Spain; elena.herasr@fjd.es (E.H.-R.); teresa.blazquezs@quironsalud.es (T.B.-S.); arantxa.torres@quironsalud.es (A.T.-R.); fernando.rengifo@quironsalud.es (F.R.-G.); claritza.caraballo@quironsalud.es (C.C.-S.); rlargo@fjd.es (R.L.); 2 Department of Nuclear Medicine, Fundación Jiménez Díaz University Hospital, 28040 Madrid, Spain; laura.landaeta@quironsalud.es (L.C.L.-K.); marta.martinezb@quironsalud.es (M.M.d.B.-A.); 3 Division of Rheumatology, Hospital Universitario de La Princesa, IIS-Princesa, 28006 Madrid, Spain; scastas@gmail.com; 4 Medicine and Psychiatry Department, University of Cantabria, 39008 Santander, Spain

**Keywords:** FDG uptake, polymyalgia rheumatica, giant cell arteritis, positron emission tomography/computed tomography (PET-CT) with 18F-fluorodeoxyglucose (FDG)

## Abstract

Polymyalgia rheumatica (PMR) is an inflammatory disease common in people aged 50 years and older. This condition is characterized by the presence of pain and stiffness involving mainly the shoulder and pelvic girdle. Besides the frequent association with giant cell arteritis (GCA), several conditions may mimic PMR or present with PMR features. Since the diagnosis is basically clinical, an adequate diagnosis of this condition is usually required. Positron emission tomography/computed tomography (PET-CT) has proved to be a useful tool for the diagnosis of PMR. The use of 18F-FDG-PET imaging appears promising as it provides detailed information on inflammatory activity that may not be evident with traditional methods. However, since PET-CT is not strictly necessary for the diagnosis of PMR, clinicians should consider several situations in which this imaging technique can be used in patients with suspected PMR.

## 1. Introduction

Polymyalgia rheumatica (PMR) is a common condition in individuals over 50 years in Western countries. PMR predominantly affects people aged 50 years and older, with an average age of onset of around 70 years [1,2,3]. It is rare in people under 50 years of age. PMR is more common in women than men, with a female/male ratio of approximately 2:1 [1,2,3].

The incidence of PMR varies geographically. In northern Europe and North America, the incidence is higher than in the other parts of the world. In this regard, in Sweden, the incidence of PMR ranges from 34 to 50 per 100,000 people aged 50 years and older per year [4,5]. In Norway, the annual incidence of PMR among residents aged 50 years and older was 112.6 per 100,000, with 137.7 per 100,000 in women and 83.2 per 100,000 in men [6]. In Olmsted County, Minnesota (USA), where the population is predominantly of Scandinavian descent, the incidence has been reported at 63.9 per 100,000 per year, with a prevalence of 701 per 100,000 [7,8]. A lower incidence was reported in southern Europe and other regions. In this regard, in Northern Spain, the incidence was 18.67 cases per 100,000 people aged 50 years and older per year between January 1987 and December 1996 [9]. In Reggio-Emilia (Italy), the average annual incidence rate of PMR was 12.8 per 100,000 population aged 50 years or older [10].

A genetic predisposition contributes to the development of PMR [11]. Certain types of human leukocyte antigens have been linked to an increased risk [12]. Moreover, an association with gene polymorphisms located outside the HLA region has also been described [13,14].

Environmental triggers, such as infections, may precipitate the onset of PMR in genetically predisposed individuals [1,3]. The exact nature of these triggers remains unclear. Some studies have suggested seasonal variations in the incidence of PMR, with higher rates of diagnosis in the winter months. This could be related to environmental factors like infections. PMR has been reported following influenza B infection and, more recently, after SARS-CoV-2 infection [15,16].

PMR is characterized by pain and stiffness in the shoulders and proximal parts of the arms, the hip girdles and proximal parts of the thighs, and the neck [17,18]. Diagnosing PMR is usually straightforward in typical patients who exhibit hip and shoulder involvement, along with elevated levels of erythrocyte sedimentation rate (ESR) or C-reactive protein (CRP). However, besides the frequent association with giant cell arteritis (GCA), several conditions may mimic PMR or present with PMR features [19]. On the other hand, in some cases, PMR can present with normal levels or with a slight increase in acute phase reactants, which makes diagnosis difficult [20]. Therefore, an adequate diagnosis of this condition is usually required.

## 2. PMR, a Condition Associated with Bursal Involvement

Arthroscopic examinations have revealed mild synovitis in the proximal joints of people with PMR. The inflammatory infiltration observed in the synovial membranes of the shoulder and other affected joints mainly consisted of macrophages and CD4 T lymphocytes [21]. However, this mild synovitis does not fully account for the musculoskeletal manifestations and diffuse pain involving the periarticular structures in patients with PMR. In this context, magnetic resonance imaging (MRI) and ultrasonography (US) investigations have identified subacromial/subdeltoid bursitis and biceps tenosynovitis, along with the synovitis of the glenohumeral joints in these individuals [17]. These observations support the idea that the involvement of extra-articular structures may contribute to the development of PMR [21,22,23,24].

In this sense, in addition to subacromial bursitis, both hip joint effusion and pelvic bursitis can also occur in PMR. Trochanteric bursitis, as well as the less common iliopsoas and ischiogluteal bursitis, have also been observed as part of the disease spectrum. [25]. In this regard, ischiogluteal bursitis is quite specific for PMR. Interspinous bursitis affecting the cervical and lower lumbar spine has been documented in patients with PMR and could potentially contribute to the neck and back pain reported by these individuals [26,27,28].

The 2012 EULAR/ACR preliminary classification criteria for PMR have included, for the first time, the use of US data whenever this information is available.

They highlight the importance of ultrasound findings showing bilateral subacromial/subdeltoid bursitis and trochanteric bursitis in diagnosing PMR in patients experiencing inflammatory pain in the shoulder or pelvic girdle [29]. The use of US enhanced the specificity of the updated 2012 EULAR and ACR criteria [30]. However, if there are no other suggestive clinical features present, isolated US abnormalities should not be sufficient to support a diagnosis of PMR [31].

## 3. Is PET-CT Useful for the Diagnosis of PMR?

For the purposes of this review, a literature search was conducted in PubMed through May 2024. The terms used for this search included “PET” or “positron emission tomography” or “FDG” or “fluorodeoxyglucose”, combined with “PMR” or “polymyalgia”.

Taking into account that the inflammation of large joints and periarticular structures is common in PMR, the visualization of inflammation in periarticular structures is of great help for diagnosis. Because of that, positron emission tomography/computed tomography (PET-CT) has proved to be a useful tool for the diagnosis of PMR [32,33]. In this regard, PET-CT uses the radioactive tracer fluorodeoxyglucose (18F-FDG), which accumulates in areas with high glucose metabolism, such as inflamed tissues. PET allows the identification of augmented metabolism in peripheral cells involved in inflammation, such as neutrophils, lymphocytes, monocytes, and macrophages. As discussed before, inflammation in PMR primarily affects the bursae, synovial joints, and tendons, and PET-CT can clearly visualize the areas of increased metabolic activity [34,35]. PET-CT can detect inflammatory changes before structural changes become apparent in conventional imaging like X-ray or magnetic resonance imaging. This early detection is crucial for initiating prompt treatment and managing symptoms effectively [36,37,38].

An important point to be kept in mind is that a wide spectrum of conditions can mimic PMR [17,39,40,41]. Among them, inflammatory arthritis, particularly late-elderly-onset rheumatoid arthritis that often presents with a polymyalgic phenotype, as well as other rheumatic conditions such as spondyloarthropathies, crystal-induced arthropathies, connective tissue diseases, or the remittent seronegative symmetrical synovitis with pitting edema (RS3PE) syndrome as well as infections and malignancies, may resemble isolated PMR [17]. Unlike these conditions, PMR presents with a well-defined periarticular inflammatory pattern that corresponds largely to the inflammation of bursal structures, which we will discuss below. PET-CT may also disclose the coexistence between PMR and GCA [40].

A few years ago, van der Geest et al. performed an extremely interesting systemic review on the diagnostic value of 18F-FDG-PET-CT in PMR. The review article included a meta-analysis that encompassed 20 studies [42]. Nine studies, encompassing 636 patients, were deemed eligible for inclusion in the meta-analysis [42]. The study confirmed a high prevalence of 18F-FDG uptake in several sites, including the interspinous bursae, hips, ischial tuberosities, shoulders, and sternoclavicular joints. The likelihood ratios for positive uptake in these areas were statistically significant, indicating their diagnostic value. The interspinous bursae showed the highest positive likelihood ratio, followed by uptake in the hips, ischial tuberosities, and shoulders [42]. However, the authors highlighted the moderate to high heterogeneity of these studies, which was attributed to variations in patient selection, scanning procedures, and interpretation criteria. Because of that, van der Geest et al. emphasized the need for standardized protocols in using 18F-FDG-PET/CT for diagnosing PMR [42].

Camellino et al. assessed the relationship between clinical symptoms and findings from FDG-PET-CT scans in patients with PMR [43]. These authors found a significant correlation between the clinical symptoms of PMR and increased FDG uptake in specific regions, such as the shoulder and hip girdles, indicating inflammation. Increased FDG uptake in vascular structures was also observed, suggesting a potential overlap with GCA, which often coexists with PMR.

The study reinforced the role of FDG-PET-CT in diagnosing and managing PMR by highlighting its ability to detect metabolic changes consistent with the disease’s clinical manifestations [43].

Kim and Kim analyzed the diagnostic accuracy of FDG-PET-CT for PMR by synthesizing data from multiple studies [44]. To investigate this, the authors conducted a systematic review and meta-analysis of the studies employing FDG-PET-CT for diagnosing PMR. Their findings indicated that FDG-PET-CT demonstrated high sensitivity and specificity in detecting PMR [44]. These results advocate for incorporating this imaging technique into PMR diagnostic protocols. [44].

## 4. How Can We Detect the Presence of Inflammation in PMR Using PET?

There are two main methods for evaluating PET scans: visual evaluation and semi-quantitative evaluation. Here are the key differences between these methods:

Visual evaluation is based on the interpretation of PET images by a radiologist or nuclear medicine physician. This method is inherently subjective, as it depends on the experience and judgment of the observer. It involves visually inspecting the PET images for areas of increased uptake, which indicate inflammation. The intensity and pattern of uptake are assessed qualitatively, often using standardized criteria or scoring systems. It provides information on the number of sites with significant uptake equal to or greater than hepatic uptake [45]. Because of that, this method is relatively easy to evaluate and does not require complex software or extensive processing. It can be performed immediately after examination and often allows visual findings to be effectively correlated with clinical symptoms and other diagnostic information, making this method valuable in routine clinical practice.

The alternative method to PET evaluation in PMR is the semi-quantitative method that involves measuring standardized absorption/uptake values (SUVs) in specific regions of interest. In this semi-quantitative analysis, the mean maximum standardized uptake value (SUVmax) is determined. Receiver operating characteristic curves are used to establish optimal cutoffs for both the visual score and SUVmax in diagnosing PMR [45]. This provides a more objective measure of inflammation by quantifying the amount of radiotracer uptake. The semi-quantitative evaluation is more reproducible than visual assessment because it is based on standardized measurements rather than subjective interpretation. This reduces variability between observers. However, it requires additional time and resources, including specialized software and expertise in quantitative image analysis. It may not be immediately available in all clinical settings. Semi-quantitative methods can provide detailed information on the extent and intensity of inflammation, which may be particularly useful in research settings or for tracking disease progression over time. In clinical practice, these methods are often used complementarily. Visual assessment can quickly identify areas of concern, while semi-quantitative analysis can provide detailed information to confirm findings and track changes. In certain scenarios, such as ambiguous cases or research studies, a semi-quantitative assessment may offer additional value by providing precise data that can influence clinical decision-making. The choice between visual or semi-quantitative assessment depends on the clinical context, available resources, and the specific needs of the patient and healthcare provider. Integrating both methods can enhance diagnostic accuracy and provide a comprehensive evaluation of disease activity. Amat et al. confirmed that both methods are useful to identify PMR among patients presenting with rheumatic conditions [45]. In this regard, these authors assessed 222 18F-FDG PET-CT scans from two hundred and fifteen patients, of which 161 scans were performed on patients diagnosed with inflammatory rheumatic diseases, fifty-seven of them with PMR. The identification of PMR based on the presence of significant uptake in at least three sites showed a sensitivity of 86% and a specificity of 85.5%. The mean SUV max cutoff for diagnosing PMR was 2.168, with a sensitivity of 77.2% and a specificity of 77.6% [45].

## 5. PET-Scores: Useful to Differentiate PMR from Other Conditions

To improve the accuracy of 18F-FDG-PET-CT in distinguishing PMR from conditions that resemble PMR, various composite scores and algorithms have been proposed based on FDG uptake at specific sites. Typically, these scores evaluate the FDG uptake at targeted regions relative to liver uptake, with uptake equal to or higher than the liver uptake considered positive. The Leuven score evaluates twelve predefined anatomical sites (cervical spinous processes, lumbar spinous processes, left and right sternoclavicular joints, left and right ischial tuberosities, left and right greater trochanters, left and right hips, and left and right shoulders) using a three-point system: 0 (no elevated FDG uptake), 1 (moderately elevated FDG uptake, but less than the average liver uptake), or 2 (intense FDG uptake, equal to or greater than the average liver uptake) [46]. A modified version, the Leuven/Groningen score, showed similar accuracy with a simplified analysis focusing on four regions (sternoclavicular joints, interspinous lumbar processes, hips, and ischial tuberosity) [47]. The Leuven/Groningen score aggregates values, resulting in a total score that spans from 0 to 14 [47].

The Besançon index is another scoring system used in the assessment of inflammatory activity in patients suspected of having PMR. It evaluates inflammation in a wider range of anatomical sites compared to the Leuven score. Unlike the Leuven score, which evaluates twelve anatomical sites, the Besançon index assesses seventeen anatomical sites. It includes regions commonly affected by PMR, such as the shoulders, hips, spine, and other large joints, which are visually graded from 0 to 3 (0: no inflammation observed; 1: mild inflammation; 2: moderate inflammation; 3: severe inflammation). A grade of two or higher at any site indicates the presence of inflammation. This scoring system assists healthcare providers in diagnosing and managing PMR effectively. The Besançon score helps clinicians quantify the extent of inflammation in patients with PMR, aiding in the diagnosis and monitoring of disease activity. A higher total score indicates more widespread inflammation, potentially guiding treatment decisions [48].

The Saint-Étienne algorithm is another diagnostic tool used for assessing inflammatory activity in patients suspected of having PMR. This algorithm focuses on only two anatomical sites: the interspinous bursa and the trochanteric bursa. The presence of inflammation is typically assessed visually [47,49,50]. Another diagnostic tool used for evaluating inflammatory activity in patients with suspected PMR is the Heidelberg. Unlike the Saint-Étienne algorithm, it assesses five anatomical sites (interspinous bursa, trochanteric bursa, glenohumeral joint, hip joint, and ischial tuberosity). Similar to the Saint-Étienne algorithm, a grade of two or more at any of the assessed anatomical sites is considered positive for inflammation [47,49,50]. Both the Heidelberg and the Saint-Étienne algorithms provide a simplified approach to diagnosing PMR based on the presence of inflammation at specific anatomical sites. They offer a standardized method for clinicians to assess inflammatory activity, aiding in diagnosis and treatment decision making. Van der Geest et al. conducted a comparison of these scores and algorithms, revealing that the Leuven score exhibited the highest diagnostic utility [47]. Furthermore, the Leuven/Groningen score, which targets four predefined regions (sternoclavicular joints, interspinous lumbar bursae, hips, and ischial tuberosities), showed similar diagnostic accuracy with the added advantage of simplified analysis [47].

Recently, Brinth et al. evaluated the established scoring systems alongside a novel simplified scoring system called the Copenhagen score. This system employs either the visual or semi-quantitative analysis of periarticular FDG uptake in the shoulders and at the ischial tuberosities [51]. For the semi-quantitative analysis, the SUV peak at the target site was compared to the mean SUV of the superior vena cava and liver, and then converted into a three-point scoring system. As previously noted by Van der Geest et al. [47], all the scoring systems, including the simplest ones focusing on a few anatomical regions, exhibited good diagnostic performance for PMR in patients not treated with glucocorticoids [51].

Ikuma et al. conducted a study to identify key sites for distinguishing PMR from rheumatoid arthritis (RA) using 18F-FDG-PET-CT. The study involved 35 patients with PMR and 46 patients with RA. Their findings indicated that FDG uptake in the shoulder joints, lumbar vertebrae spinous processes, pubic symphysis, sternoclavicular joints, ischial tuberosities, greater trochanters, and hip joints helped differentiate PMR from RA. Specifically, FDG uptake in at least one of the ischial tuberosities showed the highest diagnostic value for distinguishing between the two conditions [52].

## 6. What about Other Clinical Studies?

Several clinical studies have assessed the role of PET in patients with PMR. In this regard, Casadepax-Soulet et al. conducted a retrospective study with 85 patients with new-onset PMR and 75 controls who underwent 18F-FDG PET-CT [53]. They performed both the quantitative and semi-quantitative analyses of FDG uptake at sixteen sites. They found that the patients with new-onset PMR had a higher mean number of sites with significant FDG uptake than the controls, particularly in the hips, shoulders, and ischial tuberosity [53].

Shoulder involvement is a hallmark of PMR as confirmed by Sondag et al. [48], Henckaerts et al. [54], and Yamashita et al. [55].

We investigated the effectiveness of 18F-FDG-PET-CT in detecting PMR in patients with large vessel GCA [56]. To explore this and identify the best predictors for diagnosing PMR using 18F-FDG-PET-CT in patients with large vessel GCA, we analyzed a cohort of 54 patients diagnosed with large vessel GCA at our hospital [56,57]. We assessed nine extravascular areas, encompassing 16 potential sites: acromioclavicular and sternoclavicular joints (2 each), hips (2), shoulders (2), greater trochanters (2), ischial tuberosities (2), symphysis pubis entheses (2), and cervical and lumbar interspinous processes. Each patient was assigned a total score ranging from 0 to 16, based on the sum of all the sites demonstrating significant FDG uptake [57]. Twenty-one of the fifty four patients with large vessel GCA had been clinically diagnosed as having PMR [56]. The patients with a clinical diagnosis of PMR more commonly exhibited significant extravascular FDG uptake at the sites examined. This was particularly evident in the shoulders, where 76.2% of the patients with PMR showed significant FDG uptake compared to 24.2% of those without PMR. Other areas, such as the cervical and lumbar interspinous processes, hips, and greater trochanters, also exhibited a higher frequency of significant FDG uptake in the patients with PMR (*p* < 0.05 for all the comparisons). Consequently, the total score of significant FDG uptake (SCORE 16) was higher in the patients with large vessel vasculitis-GCA and PMR compared to those without PMR (5.10 ± 4.05 vs. 1.73 ± 2.31; *p* < 0.001). These findings indicate that the number of affected extravascular sites was greater in patients with PMR [57]. According to our data, significant 18F-FDG-PET-CT uptake in the shoulder, greater trochanter, and lumbar interspinous areas may facilitate the identification of PMR in patients with large-vessel GCA. All of these observations are in line with the recommendations put forward by van der Geest et al., who highlight the importance of evaluating multiple anatomic sites, including the shoulders, sternoclavicular joints, interspinous bursae, ischial tuberosities, hips, and greater trochanters using 18F-FDG PET-CT to detect suspected PMR. As these authors concluded, the significant FDG uptake at multiple anatomic sites is a strong indication of PMR [42,58]. In this regard, Slart et al. confirmed that 18F-FDG PET-CT is highly effective to identify inflammation in large vessels, offering precise imaging that correlates well with clinical and histopathological findings [58]. Moreover, this imaging technique was also found to be very useful for detecting inflammation in characteristic areas such as the shoulders, hips, and ischial tuberosities. Therefore, this approach is highly valuable for distinguishing PMR from other inflammatory or degenerative conditions and for assessing the extent of the disease [58].

## 7. When Do We Have to Perform 18F-FDG PET-CT?

A potential limitation of the use of 18F-FDG-PET-CT in patients with suspected PMR is that this is an expensive technique not available in many centers yet. Moreover, some authors have emphasized that the routine use of 18F-FDG-PET/CT should not be recommended in the typical cases of PMR [41]. However, these authors suggest considering 18F-FDG-PET-CT in cases where patients with PMR present atypical features, lack elevated laboratory markers of inflammation, or have a history of cancer [58]. In this regard, a diagnosis of PMR in patients presenting with normal acute-phase reactants may be a challenge [20]. This is also the case for patients with a previous history of cancer [39,40]. With respect to this, it is true that patients with cancer often present with atypical features such as a lack of morning stiffness; more widespread pain, not only located in the shoulder and hip girdles; and poor response to glucocorticoids [39]. However, there are situations where a patient previously treated for cancer may genuinely have PMR. In such cases, it is crucial to rule out cancer relapse and accurately diagnose new-onset PMR.

**Figure 1:**
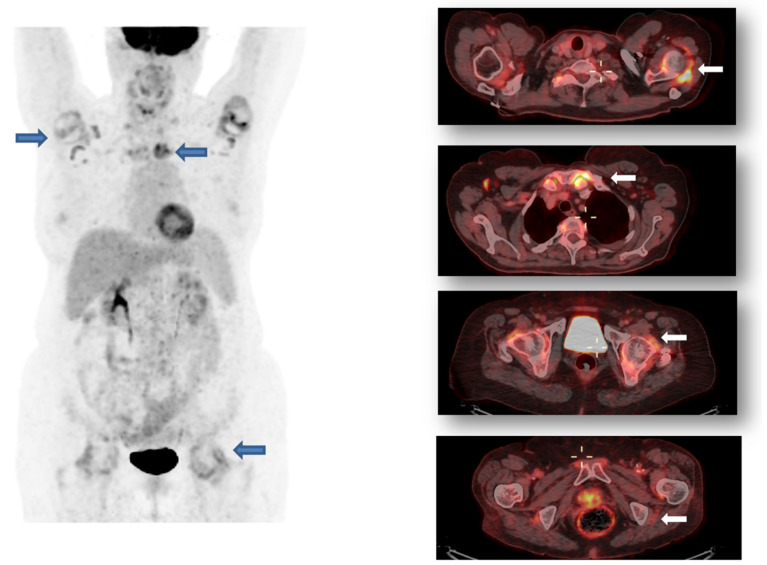
82 year old woman with significant FDG uptake around the glenohumeral, sternoclavicular, costovertebral joints, lumbar interapophyseal spaces, coxofemoral joints, ischial tuberosities, and pubis, suggestive of diffuse osteoarticular pathology with an inflammatory component, in the context associated with PMR. Blue arrows: FDG uptake around glenohumeral, sternoclavicular and coxofemoral joint. White arrows: FDG upatake around glenomeral, esternoclavicular, coxofemoral and ischial tuberosities.

Figure 1 shows the results of 18F-FDG PET-CT of an 82 year old woman diagnosed with PMR. Ten years earlier she had been diagnosed with breast cancer, treated with surgery, chemotherapy, and radiotherapy, currently in remission. PET-CT revealed increased FDG uptake in the shoulders, cervical and lumbar spine, and hips. These findings were consistent with PMR.

Interestingly, in a recent study that included 220 patients with suspected PMR and/or GCA over a two-year period, malignancy was only confirmed in seven patients (3.2%). Three of them were diagnosed with PMR concurrently with malignancy. These findings suggest that not all the patients suspected of having PMR/GCA need to undergo FDG-PET-CT systematically to rule out malignancy [59].

At this point, it is interesting to remark that the immune checkpoint inhibitors (ICIs) used in cancer patients can cause the appearance of a wide spectrum of musculoskeletal symptoms, sometimes indistinguishable from primary PMR (PMR-like syndrome) [60,61]. In these patients, PET may be useful to discriminate between primary PMR and those associated with ICI treatment. With respect to this, Vermeulen et al. suggest that ICI-PMR may show a milder course with less inflammation than primary PMR on 18F-FDG-PET-CT. As a result, patients with ICI-mediated PMR may be managed with a relatively low glucocorticoid dose, less than what is typically needed for patients with primary PMR [62].

**Figure 2:**
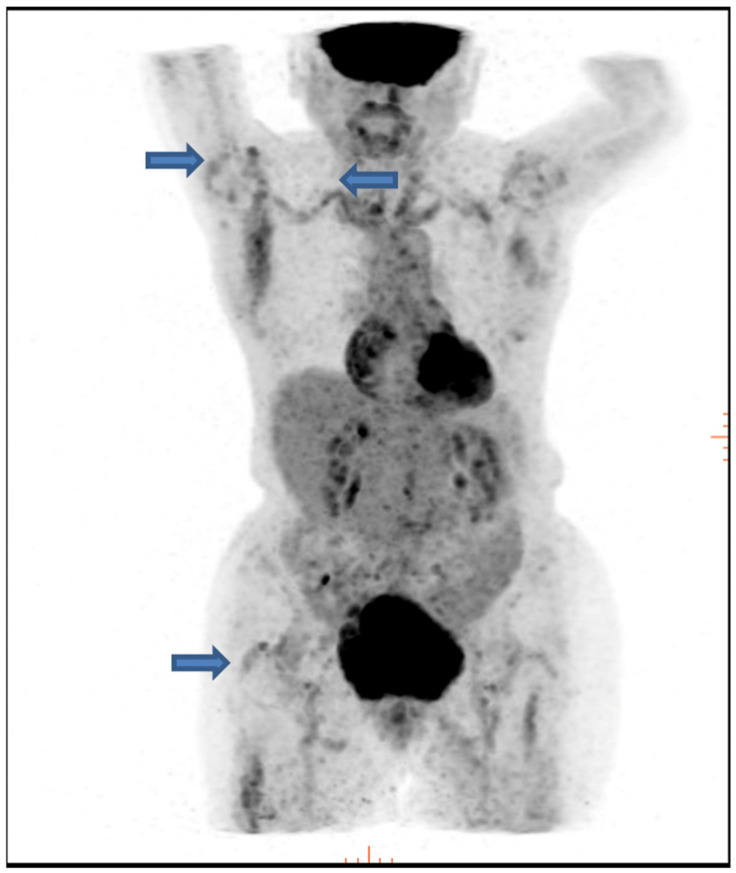
65 year old woman with significant FDG uptake around the glenohumeral and coxofemoral joints (arrows) along with large vessel vasculitis.

18F-FDG PET-CT has proven valuable in identifying patients with a predominantly extracranial pattern of GCA [63,64,65]. More than 80% of the patients with GCA demonstrate increased FDG uptake on PET imaging, particularly affecting the thoracic and abdominal aorta. Additionally, this technique can detect vascular inflammation in the lower extremity arteries of GCA patients [65,66]. GCA may initially manifest as PMR, and imaging techniques, especially PET-CT, can reveal large vessel vasculitis in at least one-third of the patients initially diagnosed with isolated PMR [17]. Figure 2 illustrates increased FDG uptake in the aorta, indicating the presence of large vessel vasculitis in a patient with PMR who did not exhibit the ischemic manifestations of GCA.

EULAR experts also suggest that FDG-PET and MRI are effective tests for detecting large vessel involvement in patients presenting with PMR or systemic symptoms where GCA is a potential diagnosis [67]. However, further research is needed to determine whether PET-CT should be routinely performed in patients with PMR. Based on our clinical experience, we recommend considering PET-CT for patients with PMR who are resistant to prednisone doses of 15–20 mg/day or who present with atypical manifestations such as predominant symptoms in the pelvic girdle [68]. In some of these cases, an extracranial large-vessel GCA may be found.

## 8. Strengths and Weaknesses of PET-CT in Patients with PMR

18F-FDG PET-CT has been shown to be highly sensitive in detecting metabolic activity associated with inflammation, allowing for the early identification of inflammatory processes in PMR. Furthermore, this imaging technique can often identify “silent” extracranial large-vessel GCA, which is particularly useful for patients with PMR with atypical symptoms such as severe inflammatory low back pain or limb claudication. In these cases, PET-CT can reveal underlying vascular problems that might not be apparent by clinical examination alone [56,68].

Since FDG-PET-CT in the typical cases of PMR shows characteristic FDG uptake patterns in areas such as the ischial tuberosity, greater trochanter, lumbar or cervical spinous processes, and scapulohumeral joints [69,70], these findings help differentiate PMR from other conditions, particularly RA [70].

Pean de Ponfilly Sotier et al. assessed the effectiveness of 18F FDG PET CT imaging in distinguishing between PMR and atypical spondyloarthritis [71]. These authors found that PET-CT images provide distinctive uptake patterns that help differentiate these two conditions, which often present with overlapping symptoms. This diagnostic tool may help clinicians make more accurate diagnoses and ultimately improve patient management and treatment outcomes.

However, there are limitations to the use of PET-CT in patients with PMR. One of them is the cost and accessibility of the technique. Furthermore, PET-CT involves exposure to ionizing radiation, which constitutes a risk, especially with repeated use. This is a crucial consideration for patients who require multiple scans over time. Furthermore, while PET-CT is sensitive to inflammation, it is not specific to PMR. Increased FDG uptake can be seen in several other conditions, including infections, malignancies, and other inflammatory diseases, which may lead to false positives and unnecessary additional testing [72].

Another limitation is the use of glucocorticoids prior to performing FDG-PET-CT. In this sense, the use of glucocorticoids may reduce the sensitivity of this imaging technique [48,73]. With respect to this, it has been found that within 3 days of high-dose glucocorticoid treatment, FDG-PET-CT can diagnose large-vessel GCA with high sensitivity [74]. However, the degree of 18-FDG uptake is reduced in patients with large-vessel GCA after 72 h of treatment with high doses of glucocorticoids, and the results may be falsely negative if the PET-CT is performed ten days after the initiation of glucocorticoids [38]. Regarding isolated PMR, some experts consider that if FDG-PET-CT is performed for diagnostic purposes, glucocorticoids should be discontinued to improve diagnostic accuracy [75]. However, other experts consider that although the effect of prednisone/prednisolone at a dose of 15 mg/day, which is the average initial dose generally used in patients with isolated PMR, may lead to a reduction in FDG uptake, the distribution of uptake on PET-CT remains consistent with that observed in patients with PMR without prior glucocorticoid therapy [38].

Another point of debate is whether PET-CT should be used in the management of PMR. There are inconclusive results regarding its correlation with relapse risk or treatment response, and changes in FDG uptakes following corticosteroid treatment have been observed, indicating challenges in its utility for long-term management. In this regard, studies on repetitive 18-FDG-PET-CT in patients with isolated PMR before treatment with glucocorticoids was initiated, and then at 3 and 6 months it was indicated that the results of FDG-PET scans in patients with PMR did not correlate with their risk of relapse [76]. In addition, current guidelines do not universally recommend its use for all patients with PMR, especially in the absence of atypical symptoms or refractory disease, due to the aforementioned factors. Further research is needed to establish clear guidelines for its use in patients with PMR.

## 9. Potential Correlation between PET-CT and Ultrasonography (US) in PMR

PET-CT is highly sensitive in detecting inflammatory changes and, as previously discussed, can help differentiate PMR from other conditions such as GCA, which often coexists with PMR. However, it has limitations due to its high cost, limited availability, and radiation exposure. In contrast, musculoskeletal US is a non-invasive imaging technique that can visualize inflammation in joints and soft tissues. In PMR, US can detect bursitis, tenosynovitis, and synovitis, particularly in the shoulders and hips. Unlike PET-CT, US is cost-effective, widely available, and does not involve radiation exposure. It can provide real-time images and is useful for guiding joint aspirations or injections. However, US also has limitations, such as being operator-dependent and less effective at assessing deeper structures compared to PET-CT.

PET-CT and US can have complementary roles in managing patients with PMR. Both techniques can identify similar inflammatory changes, though through different ways. PET-CT provides a comprehensive overview of metabolic activity, while US offers a detailed structural imaging of superficial joints and tissues. Their combined use can increase diagnostic accuracy. Both PET-CT and US can detect bursitis in the shoulders and hips, which are the key diagnostic features of PMR. While PET-CT is highly sensitive, US is more practical for routine follow-up and monitoring.

In an interesting pictorial review on PET-CT in PMR, Rehak et al. reported on the use of 18F-FDG-PET-CT in a 55-year-old woman evaluated during a relapse of PMR to rule out co-incidental malignancy. PET-CT disclosed active inflammation in the shoulders, hips, and sternoclavicular joints, as well as near the ischial tuberosities and lumbar spinous interspaces. US confirmed these inflammatory changes. These findings support a good correlation between PET-CT and US [69].

## 10. Conclusions

In daily clinical practice, most patients with PMR are managed without the need of using PET-CT. In these cases, an appropriate clinical assessment and the routine laboratory markers of inflammation can help establish a diagnosis of PMR. However, PET-CT may be useful to establish a comprehensive evaluation of PMR [77].

Since PET-CT is not required for the diagnosis of PMR, clinicians must consider several situations in which this imaging technique may be used in patients with suspected PMR.

These situations are described below (Table 1):

Patients with typical clinical manifestations in which there is a shoulder inflammatory involvement associated with other clinical manifestations such as hip involvement but who have normal markers of inflammation [20,58].

An initial dose of prednisone/prednisolone ranging between 12.5 and 25 mg/day is recommended for the management of isolated PMR [78]. Following this procedure, most patients should improve. Consequently, FDG-PET-CT may be used in a patient with typical clinical features who does not experience adequate response (a remarkable improvement of symptoms within a week) after the onset of 15–25 mg of prednisone per day.

PET-CT may be also used in patients with suspected PMR with atypical presentations. It may also be the case in patients in whom the clinical picture is confusing and does not match common diagnostic criteria [39,58].

PET-CT may help us to exclude other conditions that present with similar symptoms, such as RA, malignancies, infections, or other inflammatory diseases [35,36].

Another indication may also be patients with suspected GCA as PMR and GCA are often overlapping conditions [38,79,80]. In this regard, PET-CT can be useful in detecting large-vessel inflammation in large vessels indicative of GCA [81,82]. With respect to this, a recent study that included a meta-analysis confirmed that 18F-FDG-PET-CT is a highly effective diagnostic tool for detecting extracranial large vessel vasculitis in patients with PMR. Its high sensitivity and specificity, coupled with its ability to provide a detailed visualization of vascular inflammation, make it a valuable addition to the diagnostic of conditions [83].

In summary, the search for a reliable diagnostic test is critical in patients with PMR, in particular in those who are not well-defined. As discussed by experts in the field [84], the use of 18F-FDG-PET imaging appears promising, offering detailed insights into inflammatory activity that may not be evident through traditional methods. However, while PET imaging shows potential, it is essential to weigh its cost, accessibility, and feasibility against its diagnostic benefits. Further research and clinical validation are necessary to determine whether PET can become a standard diagnostic approach for PMR.

## Figures and Tables

**Table 1:** Situations in which we recommend performing PET-CT for identification of PMR.

Typical clinical manifestations but normal markers of inflammation
Patients with typical clinical features who do not experience adequate response to glucocorticoids
Patients with suspected PMR with atypical presentation
Suspected large vessel vasculitis indicative of extracranial GCA
To exclude other conditions that can mimic PMR (i.e., malignancies, infections, rheumatoid arthritis, and atypical spondyloarthritis)
PMR-like syndrome in cancer patients treated with immune checkpoint inhibitors (ICIs)

Abbreviations: PET-CT: positron emission tomography/computed tomography; PMR: polymyalgia rheumatica; GCA: giant cell arteritis.

## Data Availability

The data sets used and/or analyzed in the present study are available from the corresponding author upon request.
